# Transcriptomic and metabolomic analysis reveals the influence of carbohydrates on lignin degradation mediated by *Bacillus amyloliquefaciens*

**DOI:** 10.3389/fmicb.2024.1224855

**Published:** 2024-01-25

**Authors:** Xiaodan Li, Zhuofan Li, Ming Li, Jingwen Li, Quan Wang, Shuxiang Wang, Shuna Li, Hongya Li

**Affiliations:** ^1^College of Life Sciences, Hebei Agricultural University, Baoding, China; ^2^Hebei Forage Microbial Technology Innovation Center, Baoding, Hebei, China

**Keywords:** transcriptome, metabolome, carbon source, carbohydrates, lignin degradation, *Bacillus amyloliquefaciens*

## Abstract

**Introduction:**

Ligninolytic bacteria can secrete extracellular enzymes to depolymerize lignin into small-molecular aromatics that are subsequently metabolized and funneled into the TCA cycle. Carbohydrates, which are the preferred carbon sources of bacteria, influence the metabolism of lignin-derived aromatics through bacteria.

**Methods:**

In this study, untargeted metabolomics and transcriptomics analyses were performed to investigate the effect of carbohydrates on lignin degradation mediated by *Bacillus amyloliquefaciens* MN-13, a strain with lignin-degrading activity that was isolated in our previous work.

**Results:**

The results demonstrated that the cell growth of the MN-13 strain and lignin removal were promoted when carbohydrates such as glucose and sodium carboxymethyl cellulose were added to an alkaline lignin-minimal salt medium (AL-MSM) culture. Metabolomics analysis showed that lignin depolymerization took place outside the cells, and the addition of glucose regulated the uptake and metabolism of lignin-derived monomers and activated the downstream metabolism process in cells. In the transcriptomics analysis, 299 DEGs were screened after 24 h of inoculation in AL-MSM with free glucose and 2 g/L glucose, respectively, accounting for 8.3% of the total amount of annotated genes. These DEGs were primarily assigned to 30 subcategories, including flagellar assembly, the PTS system, RNA degradation, glycolysis/gluconeogenesis, the TCA cycle, pyruvate metabolism, and tryptophan metabolism. These subcategories were closely associated with the cell structure, generation of cellular energy, and precursors for biosynthetic pathways, based on a − log _10_ (P adjust) value in the KEGG pathway analysis.

**Conclusion:**

In summary, the addition of glucose increased lignin degradation mediated by the MN-13 strain through regulating glycolysis, TCA cycle, and central carbon metabolism.

## 1 Introduction

Lignocellulose is generally considered to be the most abundant and sustainable feedstock for bioenergy and bio-product manufacturing (Kumari and Singh, 2018). Lignocellulose is primarily comprised of three complex compounds, namely, cellulose, hemicellulose, and lignin, which are highly resistant to degradation; thus, efficient deconstruction of lignocellulose is difficult. As a result, there has been increasing research interest in the pretreatment of lignocellulosic biomass for further processing into high-value chemicals (Bugg et al., 2011). Among these chemical, physical, and biological treatments, microbial degradation is considered a promising solution for the conversion of lignocellulosic biomass (Gupta and Verma, 2015; Achinivu, 2018; Kumari and Singh, 2018). To date, the available studies have demonstrated that biological processing platforms can convert cellulose and hemicellulose into biofuels; however, none of these platforms can convert both sugar and lignin into biofuels or high-value chemicals, resulting in a tremendous waste of lignin biomass and a huge environmental challenge (Liu C.-G. et al., 2019).

Lignin is a highly complex branched polymer derived mainly from three monolignans: the 4-hydroxyphenyl (H), guaiacol (G), and syringyl (S) units (Mnich et al., 2017). Due to the complex bond linkages between the three aromatic units of lignin, efficient delignification and lignin conversation remain major challenges for lignocellulosic biomass utilization and environmental sustainability (Liu et al., 2022). Many microorganisms and their enzymes involved in lignin degradation have been thoroughly studied over the last few decades (Atiwesh et al., 2022), e.g., white rot fungus and its extracellular ligninolytic enzymes, such as lignin peroxidases (LiPs), manganese-dependent peroxidases (MnPs), versatile peroxidases (VPs), dye-decolorizing peroxidases (DyPs), and laccases (Lac) (Hunt et al., 2013; Rodriguez-Couto, 2017). Recently, there has been growing interest in ligninolytic bacteria due to their versatile nutrient and environmental adaptation, despite their lower lignin degradation efficiency than fungi (Huang et al., 2013). Among these bacteria, members of *Rhodococcus*, *Pseudomonas putida*, *Sphingomonas paucimobilis*, and *Streptomyces viridosporus* have shown great potential in lignin degradation (Reshmy et al., 2022). Recently, some *Bacillus* sp. strains have also been reported to be highly efficient in lignin degradation (Zhu et al., 2017; Mei et al., 2020). In our previous study, a strain of *B. amyloliquefaciens* named MN-13 (accession number of the 16S rRNA gene sequence in NCBI GenBank: KP292553) with lignin-degrading ability was isolated and stored in our laboratory. The multi-copper oxidase CotA and dye-decolorizing peroxidases from *B. amyloliquefaciens* MN-13 were expressed in *Escherichia coli,* and the roles of the corresponding recombinant proteins in the cleavage of guaiacylglycerol-beta-guaiacyl ether and the oxidization of Cα and Cβ of the aromatic intermediates to generate benzaldehydes and phenyl acetones were also demonstrated (Yang et al., 2018, 2019). These findings indicate that some strains of *B. amyloliqueficiens* exhibit strong lignin degradation performance. However, compared with fungi, lignin-degrading bacteria and their ligninolytic enzymes are much less well characterized (Wan and Li, 2012; Iram et al., 2021).

With the development in omics technology, the metabolic pathways of lignin degradation mediated by bacteria have been progressively identified. Lignin degradation mediated by bacteria involves three main steps: lignin depolymerization, degradation of lignin-derived aromatics, and target product biosynthesis (Abdelaziz et al., 2016; Wada et al., 2021; Yaguchi et al., 2021). Depolymerization of lignin into aromatics depends on the oxidation catalyzed by multiple enzymes, such as laccases, peroxidases, and additional oxidative enzymes from bacteria (Masai et al., 2007; Ragauskas et al., 2014; Rahmanpour and Bugg, 2015; Kamimura et al., 2019). As a result, aromatic intermediates derived from lignin depolymerization accumulate (Zhang and Wang, 2020), initiating further degradation of aromatic compounds through various pathways, e.g., β-ketoadipate, phenylacetic acid, and meta pathways (Ragauskas et al., 2014; Salvachúa et al., 2015; Janusz et al., 2017; Li et al., 2019; Liu et al., 2019a). The degradation products of aromatics enter the central carbon metabolism process through the tricarboxylic acid (TCA) cycle (Fuchs et al., 2011; Wells and Ragauskas, 2012; Johnson et al., 2019). These pathways contribute to the microbial conversion of various lignin-derived aromatic molecules into structure carbon and energy sources. On the other hand, carbohydrates, as common, easy-to-use carbon sources for microorganisms, and the major source of energy for living cells via glycolysis and the TCA cycle, will compete with lignin to act as the preferential carbon source because both carbohydrates and lignin are finally funneled into central carbon metabolism to provide energy for cells. This competition undoubtedly interferes with lignin degradation by bacteria. On the other hand, as reported previously, the process of lignin degradation mediated by bacteria requires extensive reducing power and energy, which is mainly generated by the glycolysis of glucose (hexose), to deal with the decomposition of polymeric structures and corresponding oxidative stress during aromatic compound catabolism (Li et al., 2019). Therefore, several studies have evaluated the co-fermentation of lignin with glucose, with the findings indicating that the addition of glucose can promote the process of lignin degradation (Linger et al., 2014; Salvachúa et al., 2015, 2020; Liu et al., 2019b). However, to the best of our knowledge, no published studies have investigated the detailed mechanism, underlying the effect of carbohydrates on lignin degradation by bacteria. Therefore, this study investigated the effect of carbohydrates on lignin degradation mediated by *B. amyloliquefaciens* MN-13 metabolomics and transcriptomics approaches. The synergistic effect of different carbon sources on lignin degradation mediated by *B. amyloliquefaciens* MN-13 was explored.

## 2 Materials and methods

### 2.1 Strains and chemicals

The *B. amyloliquefaciens* MN-13 strain was isolated from fecal samples of Simmental cattle and stored at 4°C. Alkaline lignin, prepared from needle-leaved trees and broad-leaved trees, was purchased from TCI (Shanghai, China).

Trace element solution sl-450 (per liter): 0.050 g CoCl_2_·6H_2_O, 0.0050 g CuCl_2_·2H_2_O, 0.5 g EDTA, 0.20 g FeSO_4_·7H_2_O, 0.015 g H_3_BO_3_, 0.020 g MnSO_4_·H_2_O, 0.010 g NiCl·6H_2_O, and 0.40 g ZnSO_4_·7H_2_O.

Alkaline lignin-minimal salt medium (AL-MSM, per liter): 1.0 g alkaline lignin, 3.5 g Na_2_HPO_4_·12H_2_O, 1.0 g KH_2_PO_4_, 0.5 g (NH_4_)_2_SO_4_, 0.1 g MgCl_2_·6H_2_O, 0.0235 g CaCl_2_, 50 mL trace element solution SL-450, and 950 mL H_2_O at pH 7.25.

Nutrient broth (NB, per liter): 10.0 g peptone, 3.0 g beef extract, and 5.0 g NaCl at pH 7.2–7.4.

### 2.2 The cultivation of *Bacillus amyloliquefaciens* MN-13

A single colony of *B. amyloliquefaciens* MN-13 was inoculated into NB medium and incubated at 37°C and 180 r/min. After 12 h of inoculation, the seed medium of *B. amyloliquefaciens* MN-13 was centrifuged at 5000 *g* and 4°C for 15 min to collect the cells. The cells were washed three times with sterile water, inoculated into AL-MSM (Control, CK) and AL-MSM with 2.0 g/L extra carbohydrates (glucose, cellobiose, sodium carboxymethyl cellulose, degreasing cotton, and filter paper), respectively, and incubated at 37°C and 180 r/min. The fermentation broth was withdrawn at regular intervals for the determination of cell growth and lignin removal. The fermentation broth was centrifuged to collect the cells, and the cells were washed with sterile water three times and then dried with N_2_ to determine cell growth (expressed as cell dry weight). Lignin removal was determined using the acetyl bromide spectrophotometric method with a lignin content detection kit (Solarbio, Beijing, China) (Fukushima et al., 2021).

The carbohydrate that significantly promoted cell growth and lignin removal was selected, and the effects of different concentrations of the carbohydrate in AL-MSM on the cell growth and lignin removal of the MN-13 strain were evaluated as above. AL-MSM with no extra carbohydrates was set as the control group (CK).

### 2.3 Metabolomics and transcriptomics analyses

#### 2.3.1 Growth of the MN-13 strain

A 6% (v/v) bacterial suspension of the MN-13 strain was inoculated into AL-MSM and 2 g/L glucose +AL-MSM medium, respectively, at 37°C and 180 r/min. The fermentation broth was withdrawn at regular intervals for the determination of the cell density at 600 nm (OD_600_) using a UV/VIS spectrophotometer (UV-2550PC, Shimadzu, Japan).

After 22 h of inoculation, the cells were collected and washed three times with sterile water. The cells were then snap-frozen in liquid nitrogen, and the frozen cells were stored at −80°C for untargeted metabolomics and transcriptomics analyses.

#### 2.3.2 Metabolomics analysis

In total, 100 μg of frozen cells was collected into a 2-ml centrifuge tube containing 100 mg of glass beads and 1,000 μL of 50% (v/v) methanol solution (stored at 4°C). The sample was then frozen in liquid nitrogen for 5 min. Then, the sample was homogenized with a Geno/Grinder 2010 (SPEX SamplePrep, Metuchen, NJ, USA) at 1,500 rpm for 6 min and then centrifuged for 10 min at 4°C and 12,000 rpm. The supernatant was dried under a vacuum. The residue was then re-dissolved with 300 μL of 50% methanol solution (V/V) containing 4 ppm 2-amino-3-(2-chloro-phenyl)-propionic acid. Finally, the solution was filtered through a 0.22-μm microporous membrane for LC–MS analysis.

Metabolite profiling was conducted on a Thermo Vanquish Ultra-High Performance Liquid Chromatography (UHPLC) system (Thermo Fisher Scientific, USA) coupled with a Thermo Q Exactive Focus (Thermo Fisher Scientific, USA). The HPLC conditions were as follows: ACQUITY UPLC® HSS T3 column (2.1 × 150 mm, 1.8 μm) (Waters, Milford, MA, USA); flow rate of 0.25 mL/min; column temperature of 40°C; and injection volume of 2 μL. In negative ion mode, the mobile phase was acetonitrile (solvent A) and 5 mM ammonium formate solution (solvent B). Solvents A and B were combined in a gradient as follows: 2% solvent A (0–1 min), 2–50% solvent A (1–9 min), 50–98% solvent A (9–12 min), 98% solvent A (12–13.5 min), 98–2% solvent A (13.5–14 min), and 2% solvent A (14–17 min). In positive ion mode, the mobile phase was 0.1% formic acid in acetonitrile (V/V, solvent C) and 0.1% formic acid in water (V/V, solvent D). Solvents C and D were combined in a gradient as follows: 2% solvent C (0–1 min), 2–50% solvent C (1–9 min), 50–98% solvent C (9–12 min), 98% solvent C (12–13.5 min), 98–2% solvent C (13.5–14 min), and 2% solvent C (14–20 min) (Zelena et al., 2009).

A Thermo Q Exactive Focus Mass Spectrometer with a heated electrospray ionization (ESI) source was used to acquire the MS data in positive and negative ion modes. The spray voltage was set at 3.5 kV in positive ion mode and at −2.5KV in negative ion mode. The sheath gas flow rate was 30 arb, and the aux gas flow rate was 10 arb. The capillary temperature was maintained at 325°C (Want et al., 2013). Primary full scans were acquired from m/z 81–1,000 with a resolution of 70,000. MS/MS scans of the three most abundant precursor ions were achieved through high-energy collision-induced dissociation (HCD) fragmentation at 30% normalized collision energy and were analyzed in Orbitrap at a resolution of 17,500.

The metabolites were annotated using the Human Metabolome Database (HMDB) database^1^ and the Kyoto Encyclopedia of Genes and Genomes (KEGG) database.^2^ Principal component analysis (PCA) and partial least squares discriminant analysis (PLS-DA) were performed using MetaX. The metabolites with variable importance in the projection (VIP) > 1.0 and value of *p* <0.05 were considered to be differential metabolites. Volcano plots were used to filter the metabolites of interest based on their log_2_ (FC) and log_10_ (*value of p*). The functions of these metabolites and metabolic pathways were analyzed using the KEGG database. Metabolic pathway enrichment of differentially accumulated metabolites was performed. A metabolic pathway was considered to be significantly enriched when its *p*-value was <0.05.

#### 2.3.3 Transcriptomics analysis

Total RNA was extracted from the samples using an RNeasy Mini Kit (QIAGEN, Shanghai, China) and treated with DNase I. The RNA concentration was detected with a NanoDrop 2000 Spectrophotometer (Thermo Fisher Scientific), and the RNA integrity was evaluated using a Bioanalyzer 2100 (Agilent, CA, USA). mRNA with a polyA structure was enriched using oligo (DT) magnetic beads and was then broken with fragmentation buffer into short pieces. These pieces were used to synthesize the first strand of cDNA with six-base random primers and reverse transcriptase. The second strand of cDNA was subsequently synthesized by the addition of dNTPs and DNA polymerase I. The construction of the cDNA library was performed using a TruSeq Stranded mRNA LT Sample Prep Kit (Illumina, San Diego, CA, USA). The library was sequenced by PANOMIX Biomedical Tech Co., Ltd. (Suzhou, China) on an Illumina NovaSeq 6,000 platform (Li et al., 2022). The raw data were filtered to remove reads with adapter sequences and low-quality reads in order to obtain clean reads (Bolger et al., 2014). Unigenes were obtained using Trinity software for the *de novo* assembly of the clean reads (Grabherr et al., 2011).

Gene expression levels were calculated using the expected number of fragments per kilobase per transcript per million mapped reads (FPKM) value (Roberts et al., 2011). The differentially expressed genes (DEGs) analysis was performed using DESeq (Benevenuto et al., 2019). The DEGs were identified using a threshold of *p* < 0.05 and |log_2_FC| >1. To demonstrate the expression patterns of the genes in different samples, hierarchical cluster analysis was performed using the Short Time-series Expression Miner (STEM) program (version 1.3.11). The GO and KEGG pathway enrichment analyses of the DEGs were performed using R software based on the hypergeometric distribution.

#### 2.3.4 Joint analysis of transcriptome and metabolome

The Pearson correlation analysis method was used to examine the associations between the metabolome and transcriptome. The Pearson correlation coefficient (PCC) and the corresponding *p*-value were used for screening.

#### 2.3.5 Real-time quantitative PCR (qRT-PCR) assays

The qRT-PCR assays were performed using a CFX Touch 96-Well System (Bio-Rad, California, CA, USA) to verify the expression levels of the selected genes in the transcriptomics analysis. Six DEGs were selected, and RNA was extracted using an EASYspin Plus Kit (Aidlab, China). The RNA was then reverse transcribed into cDNA using a HifiScript gDNA Removal RT MasterMix Kit (Cwbio, China). In total, 1 μL of cDNA template, 10 μL of 2× SYBR Green Master Mix (Useverbright, China), 1.2 μL of upstream and 1.2 μL of downstream primers, and 6.6 μL of RNase-Free H_2_O were added in a 20-μl reaction system. Three-step PCR reactions were performed with 95°C pre-denaturation for 120 s, 95°C deformation for 5 s, 55°C annealing for 15 s, and 72°C extension for 30 s. The total reaction circle was set to 40. The 16S rRNA gene was selected as an internal control. The primers used are presented in Supplementary Table S1. The relative expression levels of the tested genes were calculated using the 2^-ΔΔCt^ method. Three wells of each sample were used as three technical replicates, and three independent biological replicates were performed for all qPCR reactions.

## 3 Results and discussion

### 3.1 Effects of carbohydrates on cell growth and lignin removal

**Figure 1:**
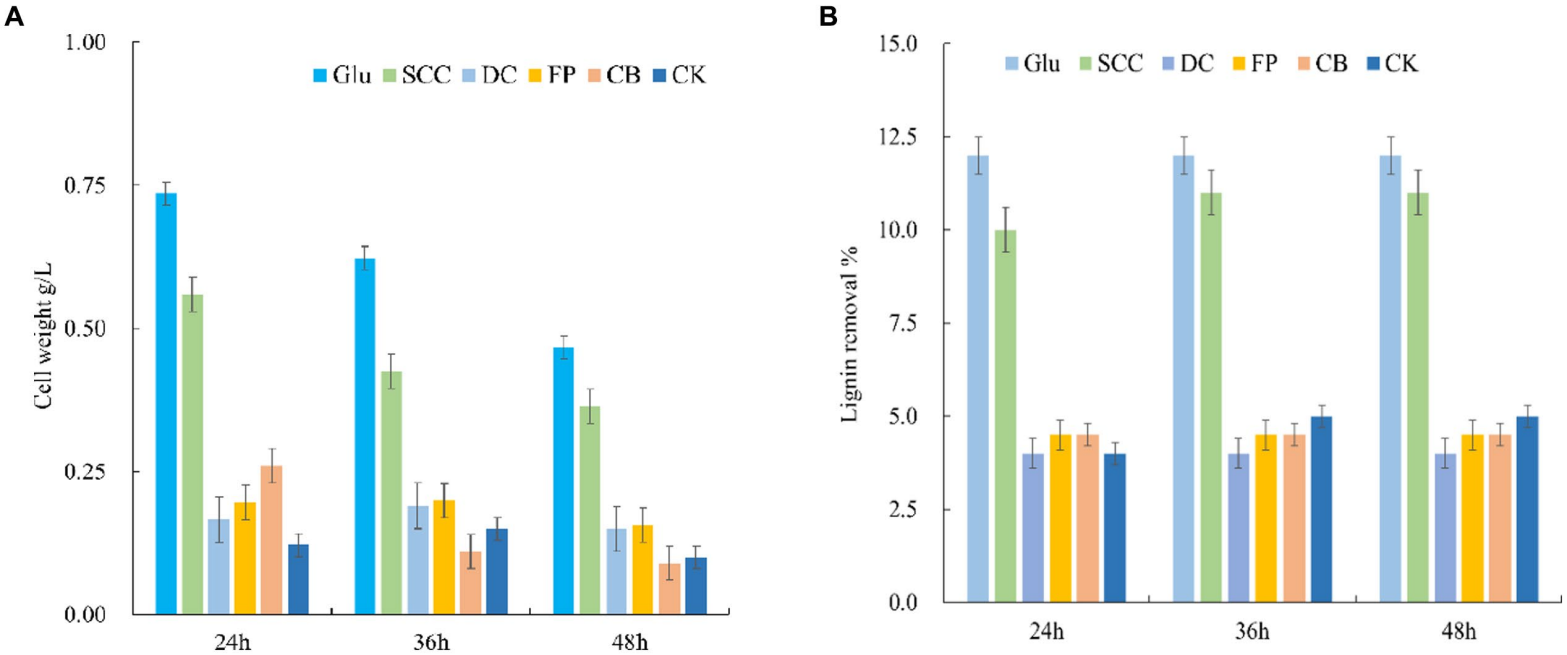
Effects of different carbohydrates on cell growth and lignin degradation. **(A)** Cell weight; **(B)** lignin removal. Glu, AL-MSM with 2.0 g/L glucose; FP, AL-MSM with 2.0 g/L filter paper; DC, AL-MSM with 2.0 g/L degreasing cotton; SCC, AL-MSM with 2.0 g/L sodium carboxymethyl cellulose; CB, AL-MSM with 2.0 g/L cellobiose; CK, AL-MSM with no extra carbohydrates.

The cell growth of the MN-13 strain and lignin removal in AL-MSM with different carbohydrates are shown in Figures 1A,B, respectively. The results revealed that the addition of different carbohydrates to AL-MSM affected the cell growth of the MN-13 strain and lignin degradation. Cell growth and lignin degradation were promoted within 24 h of the addition of carbohydrates to AL-MSM. Among the tested carbohydrates, glucose and sodium carboxymethyl cellulose had significantly greater promotion effects than other carbohydrates. When the carbohydrates were consumed, the majority of bacterial cells became dormant or formed spores, and some bacteria died. Therefore, the cell growth of the MN-13 strain decreased, and lignin removal remained unchanged over time.

### 3.2 Effects of different concentrations of glucose on cell growth and lignin degradation

**Figure 2:**
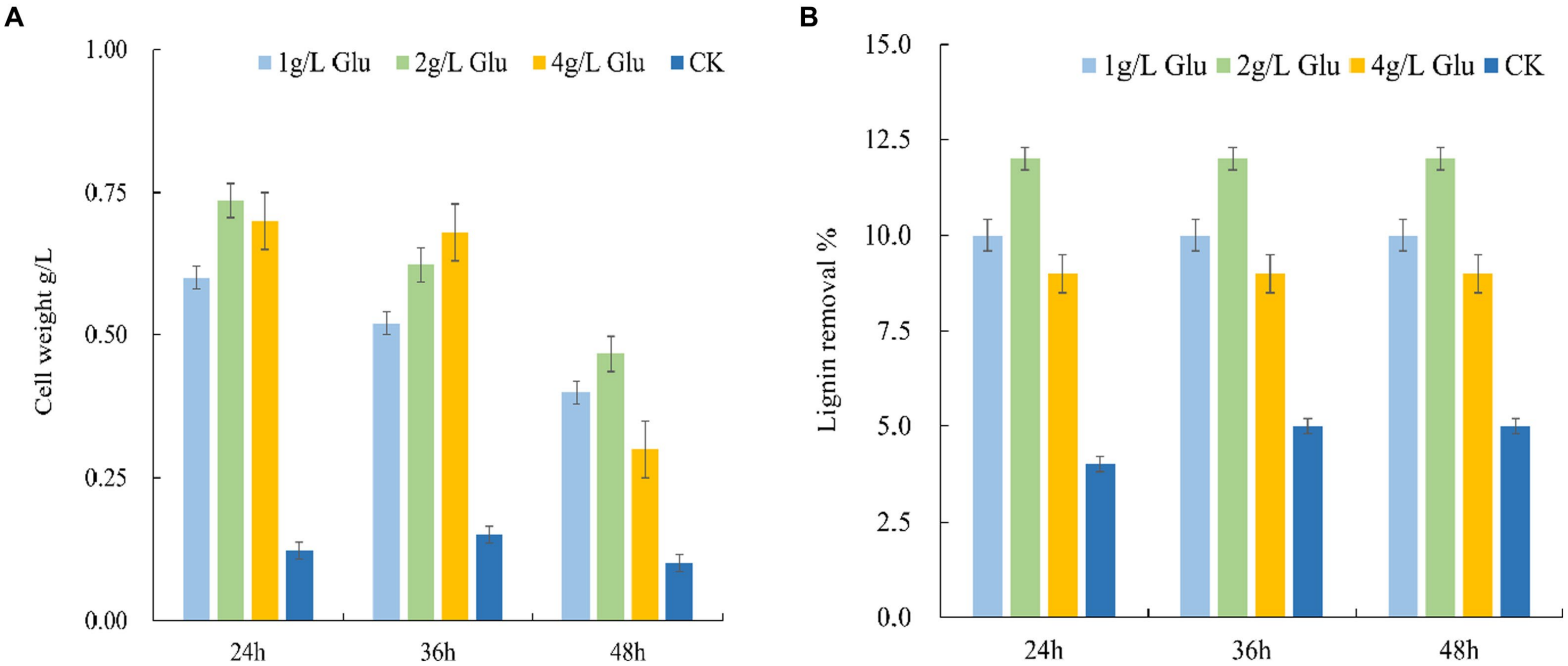
Effects of different concentrations of glucose on the cell growth of the MN-13 strain and lignin degradation. **(A)** Cell growth; **(B)** lignin removal. 1 g/L Glu, 2 g/L Glu and 4 g/L Glu: AL-MSM with 1 g/L, 2 g/L, and 4 g/L glucose respectively; CK: AL-MSM with no extra carbohydrates.

Due to the ability of glucose to promote cell growth and lignin degradation, we further investigated the effects of different concentrations of glucose on cell growth of the MN-13 strain and lignin degradation (Figures 2A,B).

There was a smaller increase in the cell weight of the MN-13 strain inoculated in AL-MSM with 1 g/L glucose as compared with the cell weight of those inoculated in AL-MSM with 2 g/L and 4 g/L glucose; however, there was no significant difference between AL-MSM with 2 g/L and 4 g/L glucose after 24 h of incubation, which suggests that 1 g/L glucose is not adequate to support cell growth, and 4 g/L might be excessive. Additionally, the maximum lignin removal mediated by the MN-13 strain within 24 h was observed with inoculation in AL-MSM with 2 g/L glucose. Therefore, 2 g/L of glucose in AL-MSM was used as the condition for the metabolomics and transcriptomics analyses.

### 3.3 Metabolomics and transcriptomics analyses

#### 3.3.1 Cell growth curves of the MN-13 strain in AL-MSM and AL-MSM with 2 g/L glucose

**Figure 3:**
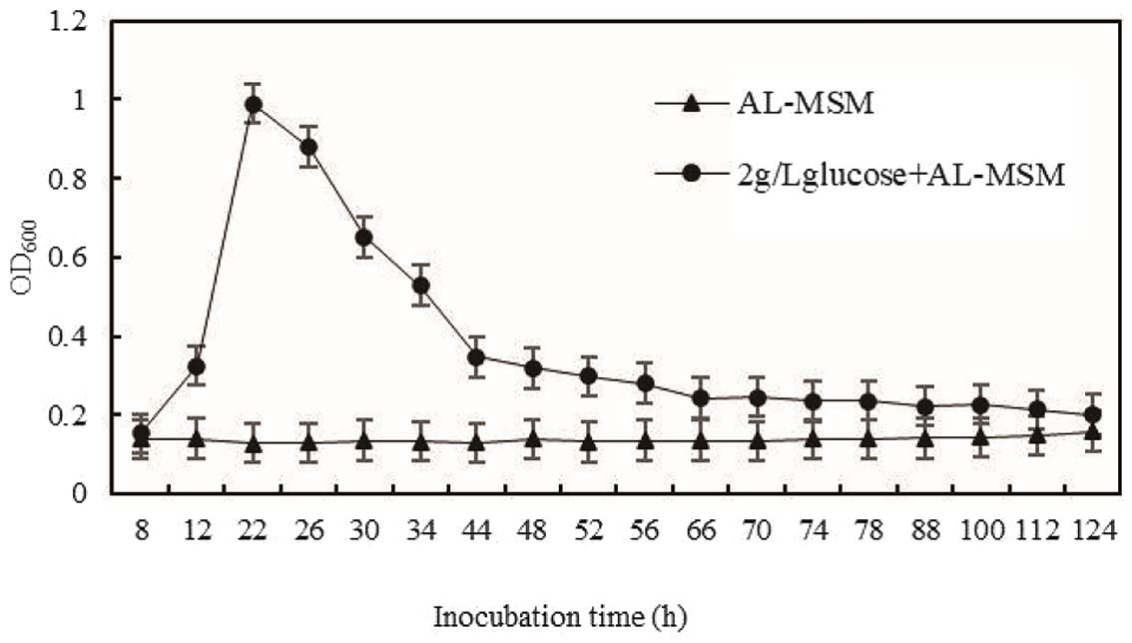
Growth curve of *B. amyloliquefaciens* MN-13 in AL-MSM and 2 g/L glucose+ AL-MSM.

To comprehensively investigate the influence of glucose on lignin degradation, the growth of *B. amyloliquefaciens* MN-13 in AL-MSM and AL-MSM with 2 g/L glucose was monitored, and the results are shown in Figure 3. The biomass of *B. amyloliquefaciens* MN-13 in AL-MSM with 2 g/L glucose reached a maximum after 22 h of inoculation while that in AL-MSM exhibited a low growth rate. Therefore, fermentation cultures were ceased at 22 h, and the cells were collected and treated for metabolomics and transcriptomics analyses.

#### 3.3.2 Metabolomics analysis

**Figure 4:**
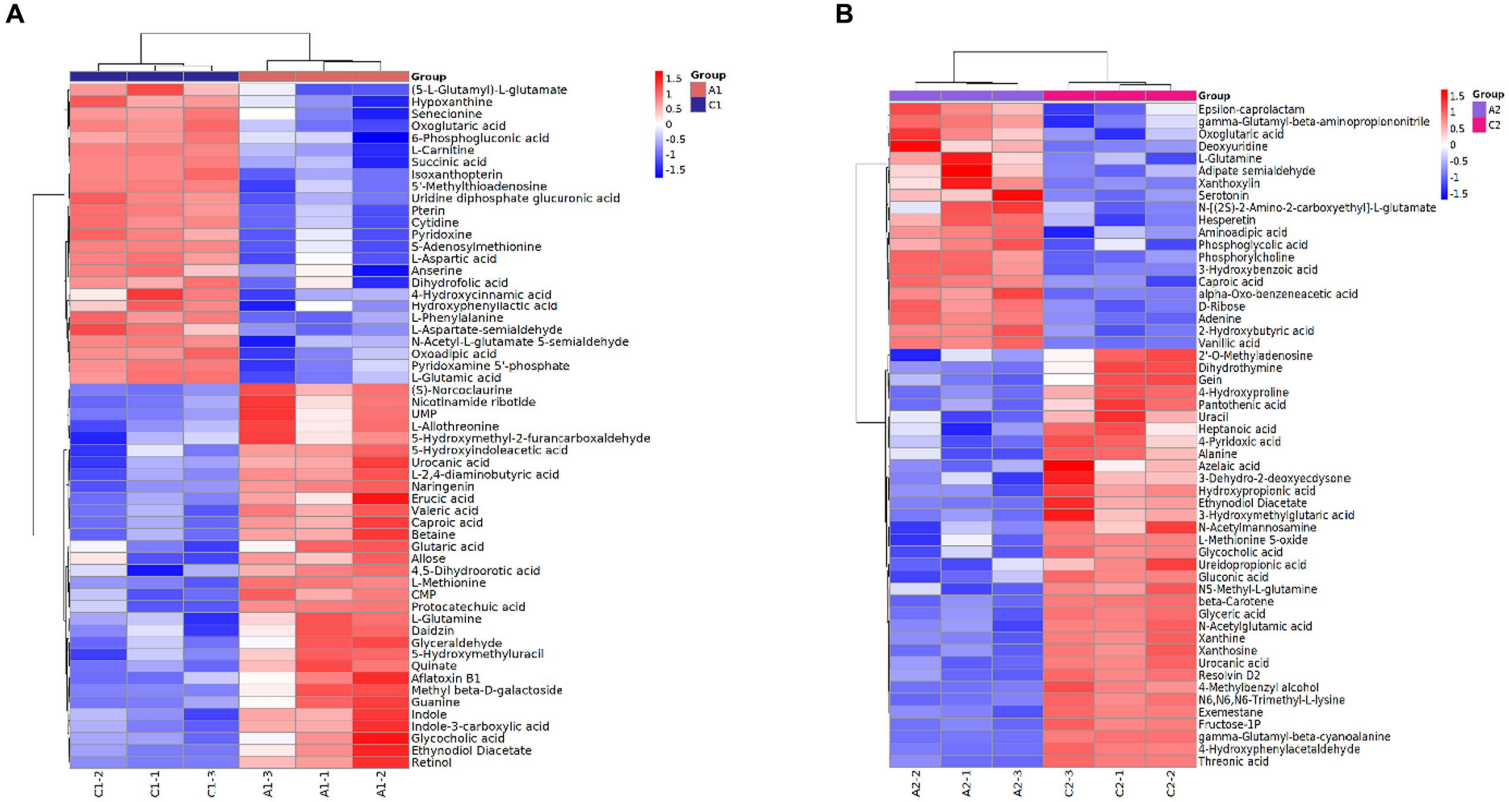
Heat map of differentially accumulated metabolites between groups A and C. **(A)** DAIMs between group A (A1) and group C (C1); **(B)** DAEMs between group A (A2) and group C (C2).

The intracellular and extracellular metabolite profiles of the MN-13 strain incubated in AL-MSM (A group) and AL-MSM with 2 g/L glucose (C group), respectively, were compared. To ensure the stability and reliability of the experimental data and results, three repetitions for each group were performed. A total of 258 metabolites were identified from the fermentation broth (extracellular), and 306 metabolites were identified from the cells (intracellular) in the A and C groups. PCA demonstrated clear grouping between the samples (Supplementary Figure S1). Based on a variable importance in the projection (VIP) > 1 and *p* < 0.05, there were 57 differentially accumulated intracellular metabolites (DAIMs) and 54 differentially accumulated extracellular metabolites (DAEMs) between groups A and C (Supplementary Tables S2, S3). These differentially accumulated metabolites (DAMs) showed different regulation patterns, with 25 DAIMs and 34 DAEMs upregulated and 32 DAIMs and 20 DAEMs downregulated between groups A and C, respectively (Figure 4). Since there were more lignin-derived aromatic compounds in DAEMs than in DAIMs (Supplementary Tables S2, S3), it could be concluded that lignin was depolymerized into low-molecular-weight aromatic compounds outside the cell, and some lignin-derived intermediates were taken up into the cells for further degradation. The latter conclusion was confirmed by the presence of the lignin-derived monomer 4-hydroxycinnamic acid (*p*-coumaric acid) and protocatechuic acid in DAIMs. Protocatechuic acid is generally considered a gradation product from lignin-derived monomer hydroxyl-cinnamic acid (Gurujeyalakshmi and Mahadevan, 1987; Andreoni et al., 1995; Parke and Ornston, 2003; Plaggenborg et al., 2003). In this study, the higher accumulation of 4-hydroxycinnamic acid and lower abundance of protocatechuic acid in group C indicates that the addition of glucose promoted the uptake of 4-hydroxycinnamic acid into the cells and the subsequent degradation of protocatechuic acid. Additionally, with the addition of glucose, oxoglutaric acid and succinic acid, involved in the TCA cycle, exhibited high accumulation, indicating greater NADH generation of energy. As reported previously, the process of lignin degradation mediated by bacteria requires extensive reducing power and energy to deal with the cleavage of the polymeric structure and corresponding oxidative stress caused by the metabolism of lignin-derived aromatics (Li et al., 2019). Therefore, it can be concluded that the addition of glucose promoted lignin depolymerization and lignin-derived aromatics uptake and catabolism, which is consistent with the findings of many previous studies (Linger et al., 2014; Salvachúa et al., 2015, 2020; Liu et al., 2019b). Meanwhile, the presence of lignin-derived monomers, such as 3-hydroxybenzoic acid, vanillic acid, alpha-oxo-benzeneacetic acid, 4-hydroxyphenylacetaldehyde, and 3-methylbenzyl alcohol in DAEMs, indicates that the addition of glucose affected the depolymerization of lignin and the uptake of these lignin-derived aromatics.

**Figure 5:**
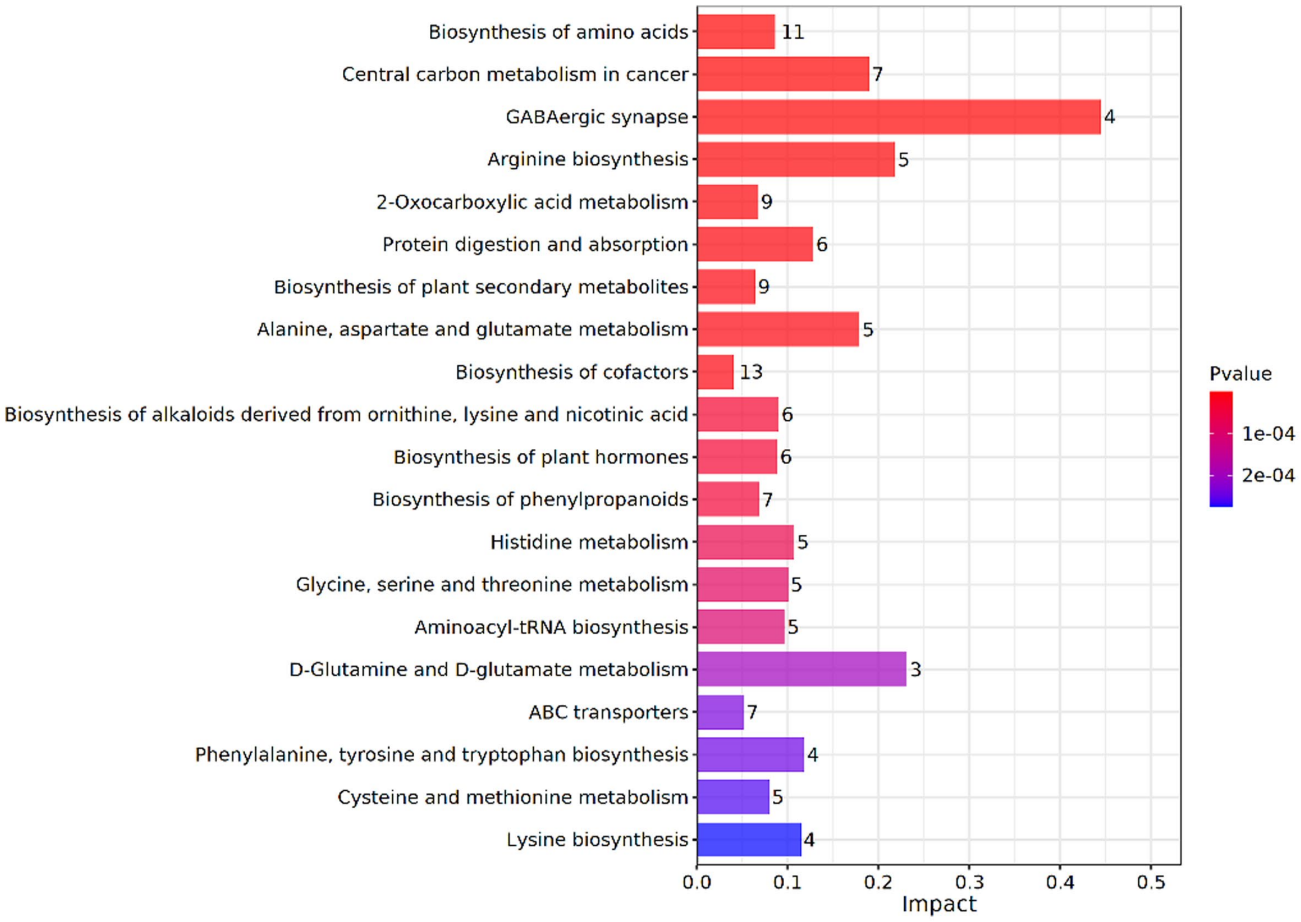
KEGG enrichment analysis of DAIMs between groups A and C. The vertical coordinates represent the metabolic pathways and the horizontal coordinates represent the effect values of enrichment in different metabolic pathways.

To further understand the effect of glucose on lignin degradation mediated by the MN-13 strain, the KEGG enrichment analysis was performed on the DAIMs. A total of 20 KEGG pathways were found in A vs. C, according to the criterion of *p*-value <0.05 (Figure 5). Among them, the DAIMs were mainly involved in the biosynthesis of amino acids; alanine, aspartate, and glutamate metabolism; central carbon metabolism; biosynthesis of phenyl-propanoids; 2-oxocarboxylic acid metabolism; phenylalanine, tyrosine, and tryptophan biosynthesis; and ABC transporters. These enriched pathways suggest that the addition of glucose promoted the degradation of lignin, which uses phenyl propanoids as building blocks and funneled lignin-derived intermediates into central carbon metabolism and the biosynthesis of amino acids.

### 3.4 Transcriptomics analysis

#### 3.4.1 Changes in the expression of genes related to lignin degradation

**Figure 6:**
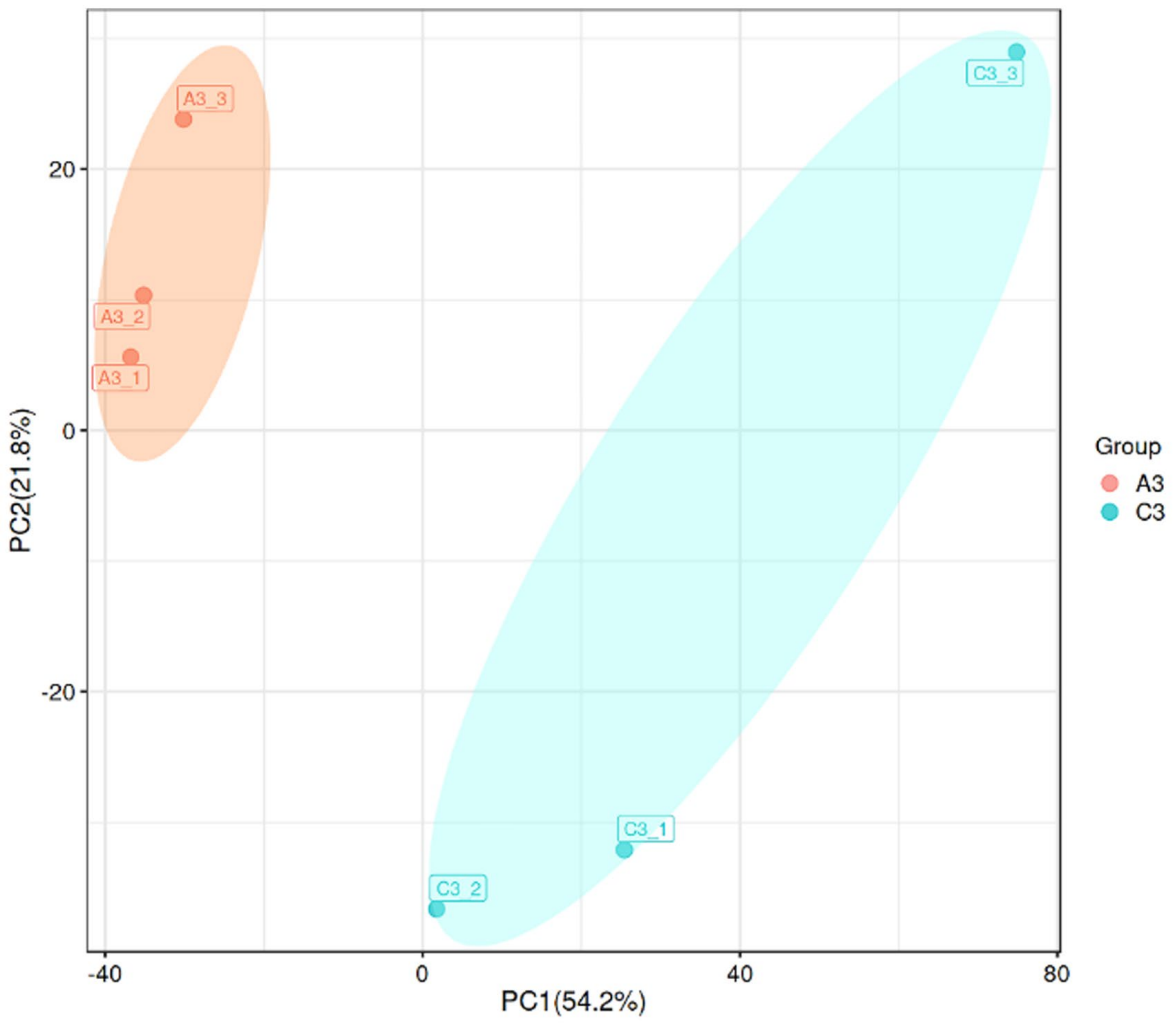
Principal component analysis (PCA) plot of the transcriptome data from the MN-13 strain incubated in AL-MSM (A3 group, red spots) and in AL-MSM with 2 g/L glucose (C3 group, blue spots).

To characterize the gene expression profiles of the MN-13 strain cultured in AL-MSM (A group) and AL-MSM with 2 g/L glucose (C group), transcriptomics analysis was performed to identify the differentially expressed genes (DEGs) between the groups (A/C). PCA indicated that six samples could be unambiguously assigned to the two groups (Figure 6). In total, 3,598 genes were identified, among which genes encoding peroxidases and key oxidases involved in lignin depolymerization, such as *gene-LUX28_RS15125* (thiol peroxidase), *gene-LUX28_RS19610* (heme peroxidase), *gene-LUX28_RS19895* (Dyp-type peroxidase YwbN), and *gene-LUX28_RS03655* (spore coat protein A, cotA laccase), were slightly upregulated in group C. This indicates that lignin degradation mediated by the MN-13 strain incubated in AL-MSM with 2 g/L glucose was more active than the MN-13 strain that was incubated in AL-MSM.

**Figure 7:**
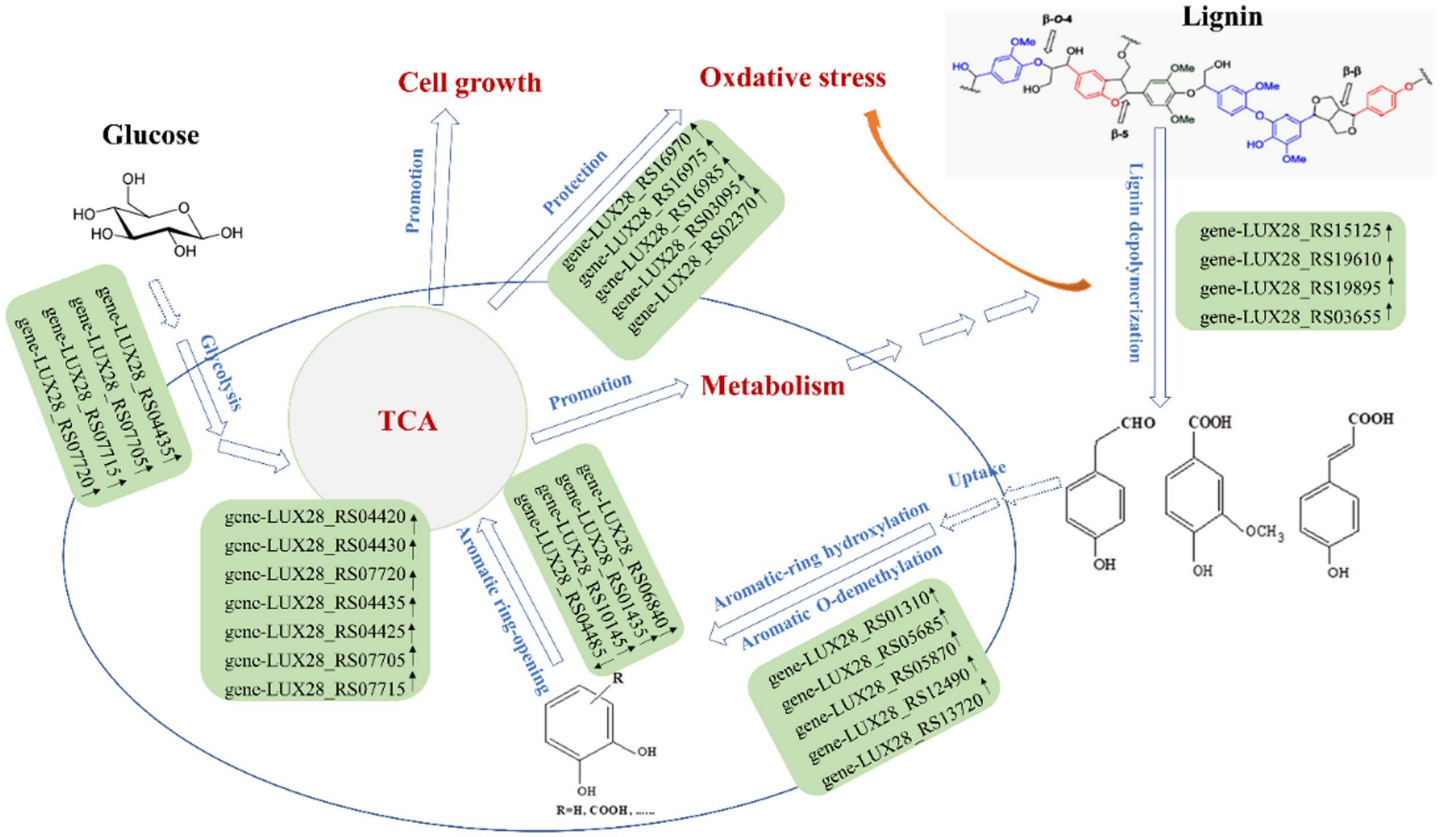
Proposed promoting effect of glucose on lignin degradation by *B. amyloliquefaciens* MN-13.

Aromatic *O*-demethylation, hydroxylation, and decarboxylation are generally considered to be rate-limiting steps in the process of conversion of lignin-derived aromatics by microorganisms (Perez et al., 2021). To date, two typical oxidative enzymes related to aromatic *O*-demethylation, that is, cytochrome P450s (P450s) and Rieske non-heme iron oxygenases (ROs), have been found to catalyze the conversion of guaiacol and 3-*O*-methyl-catechol to catechol, which is the key step in the lignin degradation process mediated by microorganisms (Bleem et al., 2022; Wolf et al., 2022). In this study, *gene-LUX28_RS05610* (Rieske 2Fe-2S iron–sulfur protein YhfW) and *gene-LUX28_RS04020* (CYPD_BACSU Probable bifunctional P-450/NADPH-P450 reductase 1) were downregulated in group C, while *gene-LUX28_RS01310* (4-hydroxyphenylacetate 3-monooxygenase) and *gene-LUX28_RS05685* (aromatic compound monooxygenase YhjG), which are considered to be catalysts for lignin-derived phenol hydroxylation (Sucharitakul et al., 2005), were upregulated (Figure 7). The pattern of downregulation of *O*-demethylation and upregulation of hydroxylation might be the result of the high accumulation of 4-hydroxycinnamic acid (*p*-coumaric acid) in cells. This finding confirms that glucose can promote the hydroxylation of p-coumaric acid, which is necessary for the ring cleavage of lignin-derived aromatics into the TCA cycle. Additionally, to date, several P450s involved in hydroxylation have been identified in natural lignin-relevant catabolic reaction pathways; however, to the best of our knowledge, they are only identified in fungal hosts (Lah et al., 2011). In this study, it remains to be confirmed whether the presence of *gene-LUX28_RS05870* (cytochrome P450), *gene-LUX28_RS12490* (cytochrome P450 YjiB), and *gene-LUX28_RS13720* (cytochrome P450 CYP102A3) in upregulated DEGs in group C means that these bacterial P450s can mediate hydroxylation. In the lower pathway, these aromatic *O*-demethylation and hydroxylation intermediates are subjected to ring opening, catalyzed by dioxygenases via the meta and ortho pathways. *Gene-LUX28_RS04485* (catechol-2, 3-dioxygenase) was downregulated, while *gene-LUX28_RS06840*, *gene-LUX28_RS01435,* and *gene-LUX28_RS10145* (putative ring-cleaving dioxygenase MhqA, MhqO and MhqE) were upregulated in group C, indicating that the addition of glucose promoted aromatic ring opening of lignin-derived intermediates.

In the 4-hydroxyphenylacetate pathway, HPA is metabolized via the meta-cleavage pathway with 3, 4-dihydroxyphenylacetate (3,4-DHPA) as the dihydroxylated intermediate and succinate and pyruvate as the final products (Ali et al., 1998). The HPA pathway has been found to play a role in the aerobic catabolism of aromatic xenobiotic compounds in several bacteria. Granja-Travez et al. found that five bacteria possess the 4-hydroxyphenylacetate (or homoprotocatechuate) pathway among 12 bacteria whose genomes were surveyed for the presence of lignin-derived aromatic degradation gene clusters (Granja-Travez et al., 2020). In the current study, the key enzymes involved in the HPA pathway, including 4-hydroxyphenylacetate-3-hydroxylase (*gene-LUX28_RS01310*), catechol-2, 3-dioxygenase (*gene-LUX28_RS04485*), and 5-carboxymethyl-2-hydroxymuconate isomerase (*gene-LUX28_RS05780*), were identified in the annotation of sequences in the transcriptomics analysis (Supplementary Table S4). Among genes encoding the three enzymes, *gene-LUX28_RS01310* was significantly upregulated in group C. Meanwhile, the comparative analysis of the metabolome revealed that DAIMs contained intermediates related to the HPA pathway, such as 4-hydroxyphenylacetaldehyde (*p* > 0.05) 3, 4-Dihydroxyphenylacetaldehyde (*p* > 0.05), pyruvate (*p* > 0.05), and succinic acid (*p* < 0.05) (Supplementary Table S5). Therefore, we speculate that the HPA pathway might be an important pathway for lignin degradation mediated by the MN-13 strain, and the HPA pathway appears to be regulated by the addition of glucose.

Additionally, lignin biodegradation mediated by bacteria involves oxidative conditions induced by hydrogen peroxide-producing enzymes, resulting in the activation of the antioxidant mechanism in bacteria (Li et al., 2019). With the addition of glucose, the expression patterns of many genes involved in oxidative stress response systems, such as catalase (*gene-LUX28_RS03095*, *gene-LUX28_RS02370*) and cytochrome bd complex (*gene-LUX28_RS17830*, *gene-LUX28_RS15730* and *gene-LUX28_RS20135*), were upregulated. Meanwhile, several studies of bacteria have shown that the SufBCD protein complex is the scaffold for iron–sulfur cluster assembly, and the Suf pathway functions as an emergency pathway under the conditions of oxidative stress (Tian et al., 2014). In the current study, upregulation of the SufBCD genes (*gene-LUX28_RS16970*, gene-LUX28_RS16975, and *gene-LUX28_RS16985*) suggests that the addition of glucose is beneficial to the protection of cells from lignin depolymerization-induced oxidative stress.

#### 3.4.2 KEGG pathway enrichment of differentially expressed genes

**Figure 8:**
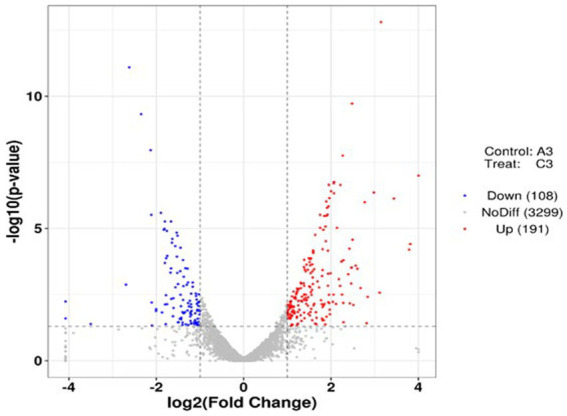
Volcano plots of DEGs in the pairwise comparisons. Volcano plot of DEGs with and without the addition of glucose.

**Figure 9:**
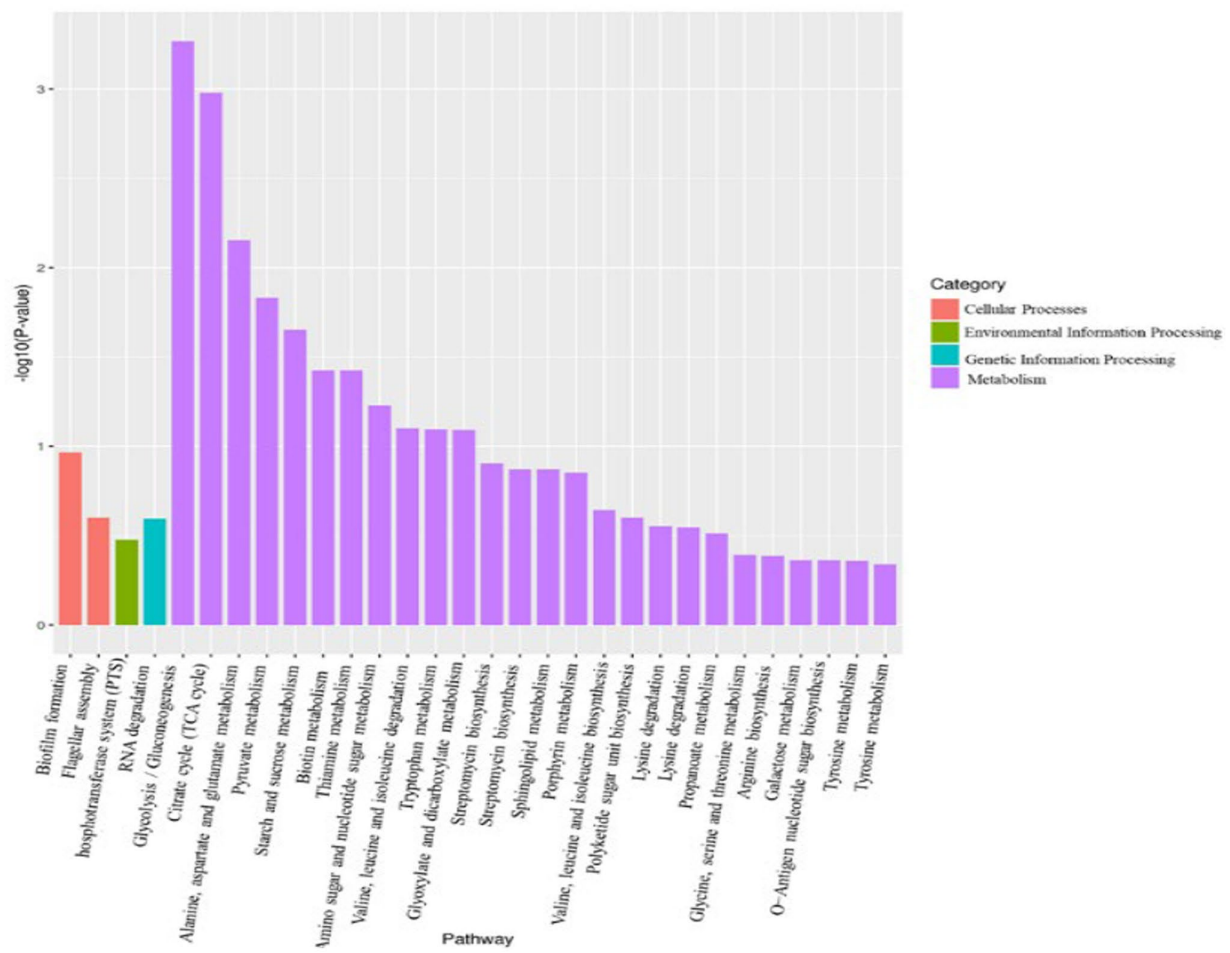
KEGG analysis of DEGs between groups A and C.

Using *p* < 0.05 and |log_2_FC| values >1 as the threshold, 299 differentially expressed genes (DEGs) were identified from pairwise comparison (A vs. C), 191 of which were upregulated and 108 were downregulated (Figure 8). To provide an overview of the effect of glucose addition on the cell transcriptome in the process of lignin degradation, KEGG pathway enrichment analysis was performed. In total, 299 DEGs with significant matches were mapped to 55 KEGG pathways (Supplementary Table S6). The addition of glucose caused the most significant changes in four subcategories, i.e., flagellar assembly, PTS system, RNA degradation, and glycolysis/ gluconeogenesis, which were assigned to four categories: cellular process, environmental information processing, genetic information processing, and metabolism, respectively (Figure 9; Supplementary Table S7). Furthermore, the DEGs were functionally enriched in the following subcategories: glycolysis/gluconeogenesis, TCA cycle, pyruvate metabolism, tryptophan metabolism, glyoxylate and dicarboxylate metabolism, propanoate metabolism, and glycerolphospholipid metabolism. These categories are closely associated with energy generation and downstream metabolism processes, meaning that the addition of glucose provided adequate energy and activated the metabolism of the MN-13 strain.

Flagellar motility enables bacteria to navigate their environment, e.g., to acquire nutrition and evade noxious substances. Increased flagellar motility in bacteria promotes biofilm initialization and loss of motility promotes increased growth rates of cells, extracellular protein excretion, and biofilm development in oligotrophic aquatic environments (Du et al., 2020). In the current study, the genes involved in the flagellar assembly and biofilm formation pathways were downregulated in group C, indicating that the addition of glucose provided a more favorable environment for cells to grow and excrete extracellular ligninolytic enzymes. This is consistent with the metabolic analysis and the increased cell growth and lignin degradation with the addition of 2 g/L glucose. Based on the KEGG analysis where the addition of glucose promoted the excretion of ligninolytic enzymes, depolymerization and modification of lignin should be more active in group C than in group A. In the process of lignin degradation, Cα-oxidative lignin-derived aromatics were generally considered to be resulted from ligninolytic peroxidases and laccase. Now a greater accumulation of these aromatic intermediates in DAEMs in A group than that of C group, indicated that there is greater extracellular activity of ligninolytic peroxidases and laccase in A group. Why were these paradoxical phenomena observed in the transcriptomic and metabolic analyses? It is well known that outer membrane vesicles (OMVs) are released by all bacteria and are loaded with a diverse array of small molecules, proteins, and genetic cargo (Cao and Lin, 2021). According to Salvachúa et al. (2020), in the study of lignin-degrading bacteria, many enzymes that were not predicted to be secreted were trafficked to the extracellular compartment via OMVs, and some ligninolytic enzymes were packaged in OMVs. Therefore, we can conclude that OMVs are the reason for the paradoxical results. That is, genes encoding heme peroxidases and CotA were upregulated with the addition of glucose in the transcriptome, but the corresponding enzymes exhibited less extracellular activities, resulting in decreased accumulation of Cα-oxidative compounds in group C. Future research will need to determine what role OMVs play in lignin degradation mediated by *Bacillus amyloliquefaciens* MN-13.

### 3.5 Validation of the DEG results by qRT-PCR analysis

**Figure 10:**
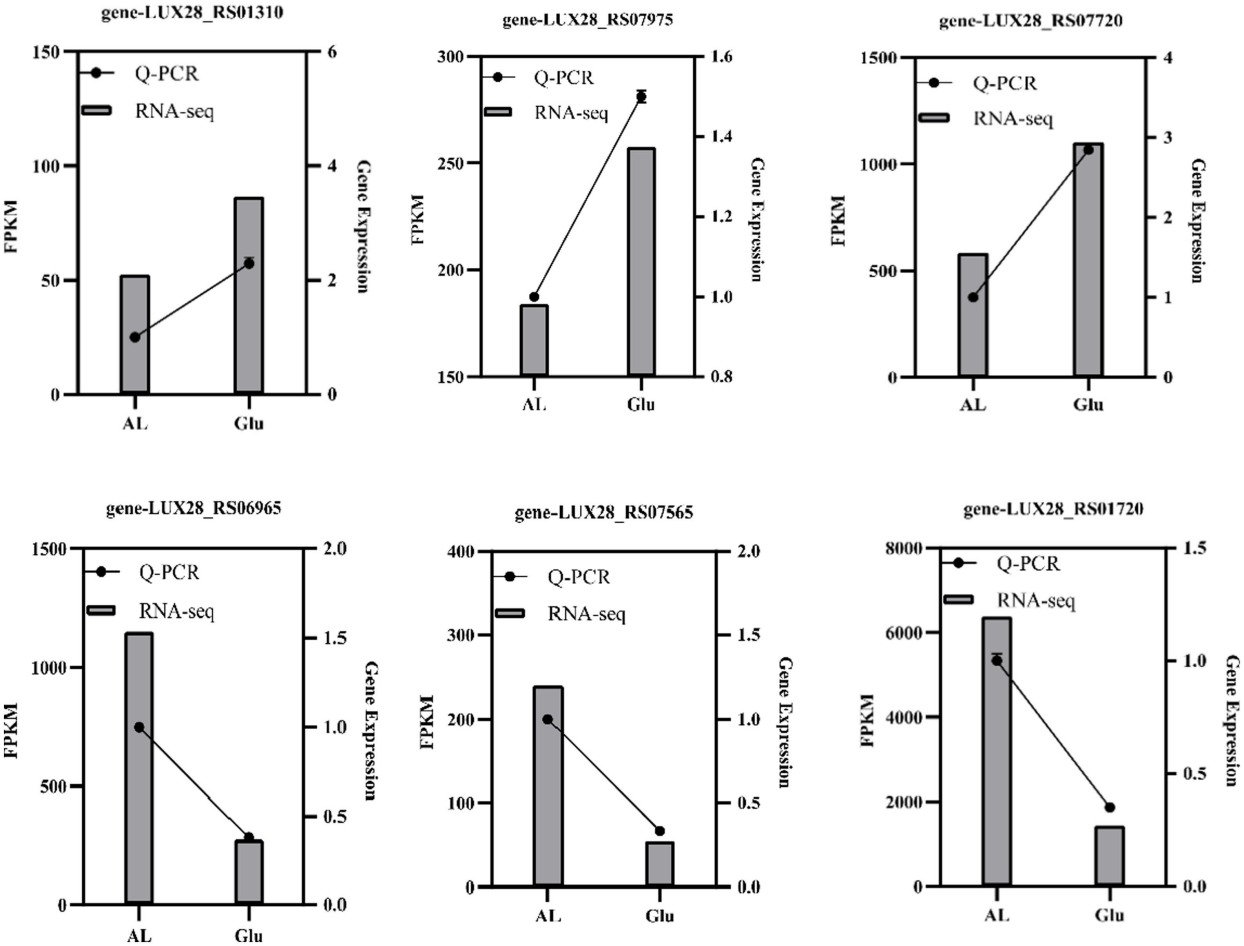
qRT-PCR validation of the expression patterns of DEGs. The left y-axis represents the expression levels of selected genes calculated by the FPKM reads method, and the right y-axis represents the relative gene expression assessed by qRT-PCR.

To verify the reliability of the transcriptome analysis, six DEGs were selected for qRT-PCR (Figure 10). The expression trends in the DEGs were largely consistent in the transcriptome analysis, although the fold changes were different. Additionally, the correlation coefficient was 0.8422, indicating a positive correlation between the RNA-seq and qRT-PCR data.

## 4 Conclusion

This study revealed that the addition of carbohydrates, such as glucose and sodium carboxymethyl cellulose, accelerates lignin degradation mediated by *B. amyloliquefaciens* MN-13 inoculated in AL-MSM, with alkaline lignin as the only carbon source. This study also provides metabolomics and transcriptomics details for exploring the synergistic effects of glucose on lignin degradation, which is mediated by bacteria. The acceleration of lignin degradation can be attributed to the upregulation of glycolysis, the TCA cycle, and central carbon metabolism with the addition of glucose. These findings offer new insights into the regulation and influence of carbon sources on lignin degradation mediated by *B. amyloliquefaciens* and a synergistic mode for sugar and aromatic metabolism. Based on these findings, a synergistic combination of cellulase-producing strains and strain MN-13 will be performed in our future research in order to verify the feasibility of microbial-mediated full component utilization of lignocellulose.

## Data availability statement

The datasets presented in this study can be found in online repositories. The names of the repository/repositories and accession number(s) can be found in the article/Supplementary material.

## Author contributions

XL: Formal analysis, Methodology, Writing the original draft. ZL: Formal analysis, Methodology. ML: Methodology. JL: Formal analysis. QW: Data curation. SW: Investigation. SL: Review and editing. HL: Conceptualization, Funding acquisition, Supervision, Review and editing. All authors contributed to the article and approved the submitted version.

## Footnotes

^1^ http://www.hmdb.ca/

^2^ https://www.genome.jp/kegg/pathway.html
