# Supplementary material for: Transcriptomic and metabolomic analysis reveals the influence of carbohydrates on lignin degradation mediated by Bacillus amyloliquefaciens
Source: Front Microbiol. 2024 Jan 25;15:1224855. doi: 10.3389/fmicb.2024.1224855 (PMC10850570; doi:10.3389/fmicb.2024.1224855)
Supplement: Supplementary file 1 [file Data_Sheet_1.zip › Table S1, Supplementary figures and material.docx]

**Supplementary Material:**

**Transcriptomic and metabolomic analysis reveals the influence of carbohydrates on lignin degradation mediated by *Bacillus amyloliquefaciens***

**Captions**

Fig. S1 Principal component analysis plots in the metabolome of group A and C.

Fig.S2 Heat map of correlation between DEGs and DAIMs.

Table S1 Primers used for the qRT-PCR genes.

Table S2 Differentially accumulated intracellular metabolites (DAIMs) between group A and C.

Table S3 Differentially accumulated extracellular metabolites (DAEMs) between group A and C.

Table S4 Differentially expressed genes (DEGs) between group A and C.

Table S5 Total metabolites between group A and C.

Table S6 KEGG enrichment result of DEGs between group A and C.

Table S7 GO enrichment result of DEGs between group A and C.

**Table S1**

Primers for qRT-PCR

| **Name** | **Primer sequence (5’-3’)** | |  | |  |
| --- | --- | --- | --- | --- | --- |
| RS07720-F | | CGCCGTCCGAACACTGATGA | |  | |
| RS07720-R | | GTGGAGGTCCGTCAATGATGTCA | |  | |
|  | |  | |  | |
| RS07975-F  RS07975-R  RS01310-F  RS01310-R | | GTGCGACCGAATGCGAACTG  GCGAATGAATCTTCTTGCTCTTCTG  TGACATCAGAGGAGACCTTGACCAA  CCATGCGAGGCGGAACACTT | |  | |
| RS01720-F  RS01720-R  RS06965-F  RS06965-R  RS07565-F  RS07565-R  16srRNA-F  16srRNA-R | | CGGATACGGCGGAAGCGATT  CCACGGCGGAATAACAACTGTCA  GGCGGACGAGCAGGACATAT  CCGAAGCAGGCAGCATATCC  TTGTCGGTTCTGCGGTAGCG  CAGCGGATGGTTCGTAATGAATGC  GAAGTCGTAACAAGGTAG  CAAGGTCTTATATTCCGTTA | |  | |

Fig. S1

A

B


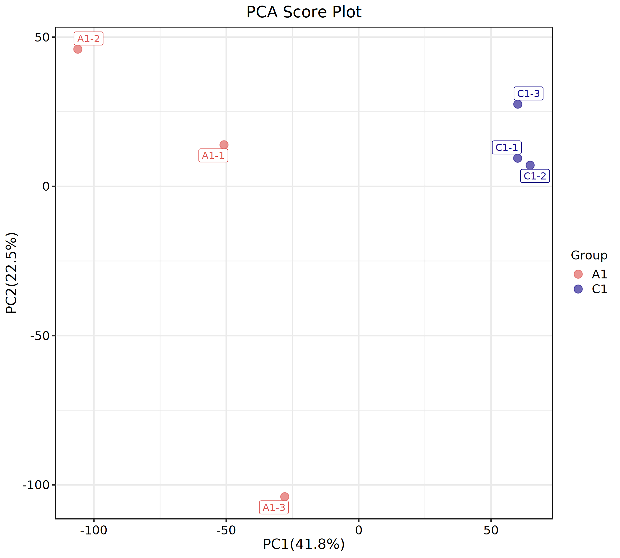

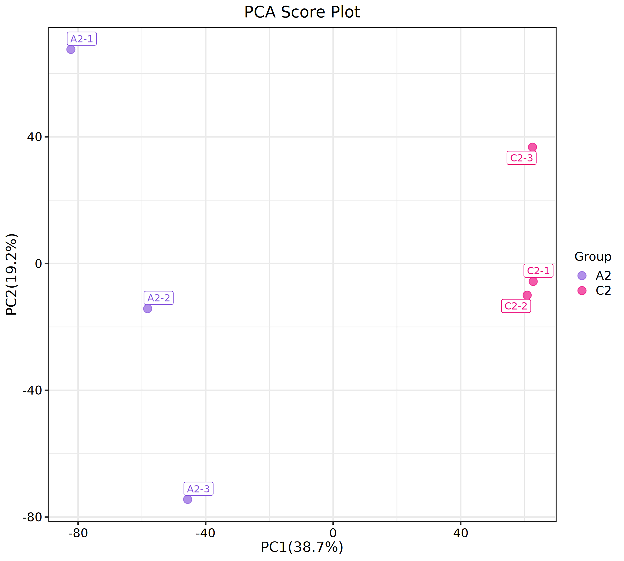


Principal component analysis plots in the metabolome of groups A and C. A: Principal component analysis plots of intracellular metabolomes in groups A and C; B: Principal component analysis plots in the extracellular metabolome of groups A and C

**Method for joint analysis of transcriptome and metabolome**

The Pearson correlation analysis method was used to examine the associations between the metabolome and transcriptome. The Pearson Correlation Coefficient (PCC) and the corresponding P-value were used for screening.

**Results of Correlation analysis**

To further understand the effect of glucose on lignin degradation by *B amyloliquefaciens* MN-13, a correlation analysis between DEGs and DAIMs was performed (**Supplementary Fig. S2**). 4-hydroxy cinnamic acid was positively correlated with genes involved in the TCA cycle, such as *gene-LUX28_RS04435* (Dihydrolipoyl dehydrogenase) (correlation coefficient 0.9422), *gene-LUX28_RS07705* (Pyruvate dehydrogenase E1 component subunit alpha) (0.6705), *gene-LUX28_RS04425* (acetoin dehydrogenase E1) (0.9328), *gene-LUX28_RS10165* (Aryl-phospho-beta-D-glucosidase BglA) (0.7071), *gene-LUX28_RS15160* (putative acyl-coenzyme A synthetase) (0.8369) and, *gene-LUX28_RS07720* (dihydrolipoamide dehydrogenase) (0.7559), while protocatechuic acid was negatively correlated with these genes (coefficients of 0.9534, 0.7239, 0.9768, 0.6090, 0.9585 and 0.8575, respectively). These results further verify that the addition of glucose promoted the uptake of 4-hydroxycinnamic acid into cells and the metabolism of protocatechuic acid, was subsequently funnelled into the TCA cycle.

Further, *gene-LUX28_RS16970* (SufB) and *gene-LUX28_RS 16985* (SufD) encoding Fe-S cluster biosynthesis proteins were also positively correlated with 4-hydroxycinnamic acid (0.9356) and succinic acid (0.9907). This indicates that the addition of glucose promoted the response of cells to oxidative stress resulting from lignin depolymerization and the further degradation of lignin-derived intermediates mediated by the MN-13 strain. This is consistent with the findings of the GO analysis (Table S7).

Finally, it should be noted that *gene-LUX28_RS01310* encoding 4-hydroxyphenylacetic acid-3-monooxygenase was significantly upregulated and the 4-hydroxyphenylacetate pathway was identified as a lignin-derived intermediates degradation pathway in the transcriptomic analysis. According to the correlation analysis, *gene-LUX28_RS01310* was positively correlated with 4-hydroxycinnamic acid (0.5745), succinic acid (0.3558) and oxoglutaric acid (0.4403). The correlation mode of *gene-LUX28_RS01310* indicates that the addition of glucose facilitated the MN-13 mediated biotransformation of lignin-derived 4-hydroxycinnamic acid into the TCA cycle via the 4-hydroxyphenylacetic acid pathway. On the other hand, the negative correlation between *gene-LUX28_RS01310* and protocatechuic acid (0.5075) suggests that there might be competition between the 4-hydroxyphenylacetic acid pathway and the protocatechuic acid pathway for the degradation of 4-hydroxycinnamic acid mediated by *B. amyloliquefaciens* MN-13.

**Fig. S3**


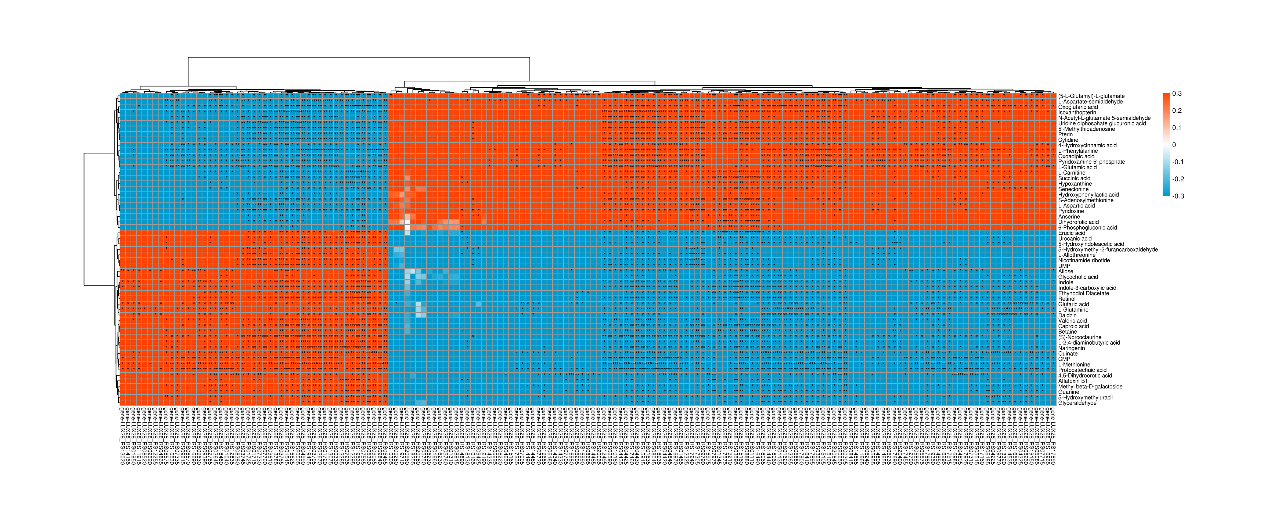


Heat map of correlation between DEGs and DAIMs. * *P*＜0.05, ***P*＜0.01, ****P*＜0.001.
